# Mobile Robot Path Planning Using Ant Colony Algorithm and Improved Potential Field Method

**DOI:** 10.1155/2019/1932812

**Published:** 2019-05-06

**Authors:** Guoliang Chen, Jie Liu

**Affiliations:** School of Mechanical and Electronic Engineering, Wuhan University of Technology, Wuhan 430070, Hubei, China

## Abstract

For the problem of mobile robot's path planning under the known environment, a path planning method of mixed artificial potential field (APF) and ant colony optimization (ACO) based on grid map is proposed. First, based on the grid model, APF is improved in three ways: the attraction field, the direction of resultant force, and jumping out the infinite loop. Then, the hybrid strategy combined global updating with local updating is developed to design updating method of the ACO pheromone. The process of optimization of ACO is divided into two phases. In the prophase, the direction of the resultant force obtained by the improved APF is used as the inspired factors, which leads ant colony to move in a directional manner. In the anaphase, the inspired factors are canceled, and ant colony transition is completely based on pheromone updating, which can overcome the inertia of the ant colony and force them to explore a new and better path. Finally, some simulation experiments and mobile robot environment experiments are done. The experiment results verify that the method has stronger stability and environmental adaptability.

## 1. Introduction

Nowadays, one of the prime concerns of mobile robot is path planning. A path planning optimization method is proposed to calculate the shortest collision free path from source to destination by avoiding static as well as dynamic obstacles. Therefore, it is necessary to select an appropriate optimization technique for optimization of paths [1, 2]. Concerning this problem, scholars proposed a lot of path planning methods, including APF [3], grid method [4], and ACO [5] and some intelligent methods, like fuzzy logic [6], neural network [7], genetic algorithm [8], artificial bee colony (ABC) [9], and particle swarm optimization (PSO) [10]. Among these methods, grid method can find an optimal path, but its efficiency is affected by environment and grid density. Neural network is good at learning, but its net structure is large, and its neuron thresholds change with time under the condition of multiobstacle and dynamic environment. Genetic algorithm has a good global search capability, but its search space is large, and its model is needed to reestablish with environment changing. ABC has the advantages of simple calculation, few parameters, and easy implementation. But, ABC also has some disadvantages such as a slow convergence. In addition, ABC is first applied to solve the problems of function optimization, while the path planning of mobile robot is a combinatorial optimization problem. Therefore, there are some difficulties in algorithm construction. PSO has a rather fast speed of approaching the optimal solution, which is an advantage to solve optimization problems of continuous systems. But, PSO also has some deficiencies, including the premature convergence and poor local optimization capability.

APF is a popular approach for solving path planning problems because of its simplicity, fast execution time, and applicability to mobile robot, unmanned aerial vehicles. Li et al. [11] proposed an innovative yaw angle-based APF function to achieve the desired road trajectory within certain road boundary and minimize the yaw angle change rate of the autonomous electric vehicle. Rasekhipour et al. [12] developed a path planning controller based on APF for Autonomous Road Vehicles which was able to consider any PF for obstacles and road structures while calculating the optimal path. Malone et al. [13] utilized the APF framework for runtime planning and leveraged a method of stochastic reachable sets to generate accurate potential fields for moving obstacles. APF easily traps into a local optimum, which leads the robot to be into a deadlock because of its zero resultant force. Many improved algorithms are developed to fix these defects of APF. Sun et al. [14] proposed an improvement based on a distance factor and jump strategy to solve the various situations of the target unreachable problem of the traditional APF. Liu et al. [15] introduced a critical value based on the distance between the robot and the obstacle to modify the repulsion direction for avoiding the local minimum point. Some scholars attempt to combine the intelligent algorithm with APF to eliminate the defects of APF. Pan et al. [16] used the fuzzy controller to improve APF method and safeguarded the reliability of the path planning and path smoothness.

ACO is an intelligence-optimized algorithm that simulates the heuristic mechanism of the shortest route based on pheromone in the process of ants foraging for food [17]. ACO has been used in mobile robot's path planning. Akbarimajd and Hassanzadeh [18] presented a path planning method for mobile robots based on two-dimensional cellular automata, which can be applied for environments with both concave and convex obstacles and be appropriate for multirobot problems as well as dynamic environments. Montiel-Ross et al. [19] developed the navigation software called Ant Colony Test Center. This software based on ant colonies is able to achieve path generation, path planning, and virtual path tracking at once. You et al. [20] designed a new dynamic search model for path planning problem of mobile robot, which increases the diversity of the ant population by using a bigger parameter in the prophase and accelerated convergence by using a smaller parameter to adjust the attenuation model in the anaphase. However, ACO has some problems such as the premature convergence and ant colony lost. For the inherent shortcomings of ACO, there are many improved methods that can be classified into improvements based on single classical ACO and improvements based on mixed algorithms combined ACO and other intelligent algorithm. Liu and Hu [21] proposed the multigranularity pattern ant colony algorithm, in which ants had different window sizes to enhance the diversity of the ants. The proposed algorithm was used for UAV path planning problem. Wang et al. [22] applied genetic algorithm to optimization and configuration parameters of the basic ant colony algorithm. Hsu and Juang [23] proposed a multiobjective, rule-coded, advanced, continuous-ant-colony optimization algorithm that was applied to design the fuzzy controller of wall-following control for a mobile robot.

Of course, ACO has more applications than just robot path planning, and there are other metaheuristic methods that can be used for reference or application in robot path planning. Chu and Tsai [24] proposed a new optimization algorithm of cat swarm optimization (CSO), and CSO is generated by observing the behavior of cats and composed of seeking mode and tracking mode by simulating the behavior of cats. The proposed CSO somehow belongs the swarm intelligence, which may give much better performance than PSO with a weighting factor. The studies deserve sustained attention. For numerical optimization problems, Tsai et al. [25] proposed an enhanced ABC optimization algorithm, in which the onlooker bee is designed to move straightly to the picked coordinate indicated by the employed bee and evaluates the fitness values near it in the original ABC, and the concept of universal gravitation is introduced into the consideration of the affection between employed bees and the onlooker bees. Aiming at the weaknesses of computational swarm intelligence such as particle swarm optimization (PSO), Ever [26] utilized simplified swarm optimization (SSO) to enhance the path planning of mobile robot in working environment with irregular obstacles, which driven from an inspiration of communal behavior of birds flocking and fish schooling.

APF is a typical online algorithm for robot path planning and has a simple and beautiful mathematical description. So, APF is still very attractive in robot path planning, although it has some inherent limitations and no learning ability. ACO uses all paths of the entire ant colony to describe the solution space of an optimization problem, and obtains the best path through positive feedback based on pheromone. The idea of ACO directly maps the mobile robot's path optimization problem, which is easy to understand and has very intuitive results. Based on the above reasons, this paper combines APF and ACO to research the problem of mobile robot's path planning under the condition of known robot's motion environment described by grid map and proposes a path planning algorithm of ACO mixed with an improved APF. The proposed algorithm can provide an initial path obtained by an improved APF to be the inspired factors of ACO for avoiding its premature convergence and local optimum.

This paper is organized in the following manner: Section 2 presents the basic approach of building a grid map of robot's environment and describes the problem of robot path planning based on the grid map.  Section 3 proposes some improved methods about APF after analyzing the existing defects of APF applied to the grid map.  Section 4 develops some improved methods about transition algorithm of ant colony.  Section 5 demonstrates results of the proposed mixed path planning method by computer simulation experiments and mobile robot's motion experiments in real environments. Finally, the contributions of this paper are summarized in Section 6.

## 2. Rasterizing Environment Map and Problem Description

The work space of robot is set to a limited area on a two-dimensional plane. The coordinates of obstacles edge points can be determined by processing the original environment map. If we expand the size of the obstacles to a safe distance (usually taken as mobile robot's radius) and add the size of the mobile robot to the obstacles, the robot can be seen as a scale-free particle, and the grid granularity can be calculated as

(1) $$\begin{array}{c} D_{\text{g}}=\phi D_{\text{r}}+\frac{\varphi }{D_{\text{m}}}+\lambda D_{\text{s}}, \end{array}$$

where *D*_g_ represents the grid size; *ϕ*, *φ*, and *λ* are the weighing coefficients; *D*_m_ is the map size; and *D*_r_ and *D*_s_ is mobile robot's size and safe distance.

In this paper, the size of the environment map is *M* × *N* (pixel unit), and then the robot environment can be divided into [*M*/*D*_g_] × [*N*/*D*_g_] ([·] means integer arithmetic) grid units.

**Figure 1:**
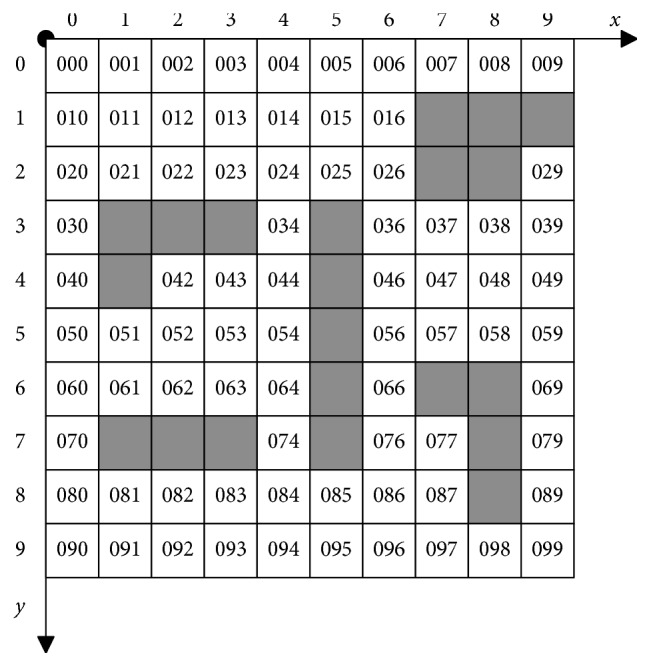
Typical grid model.

Grids are numbered from left to right and from top to bottom. Meanwhile, grids are assigned respectively to distinguish whether the grid is an obstacle space or a free space. The values of grid units are decided by the state value of these pixels. The grid is an obstacle grid if the amount of pixel with value 0 exceeds 1 in the area of the grid and defined as 0 or 1. Eventually, the typical grid model is obtained as shown in Figure 1.

The problem of mobile robot path planning can be described to find an obstacle-free shortest path from the starting grid to the target gird. In Figure 1 calculated by equation (1), the mobile robot considered as a scale-free particle adopts the movement form of linear octree and reaches the neighbor grids along the top, right, bottom, left, upper right, lower right, lower left, and upper left eight directions.

## 3. Improved APF

### 3.1. Definition of APF

Attractive field is the effect of the target point on the robot, and it can guide the robot to move to the target point. Attractive field is radial distribution about the target point and the direction of attraction points to the target point. The traditional attractive potential function is defined as

(2) $$\begin{array}{c} U_{\text{attr}}\left(X_{\text{r}}\right)=K\times {\left(\left|X_{\text{r}}-X_{\text{g}}\right|\right)}^{\alpha }, \end{array}$$

where *K* is a constant, **X**_r_ represents the robot's current position coordinates, **X**_g_ represents the target point coordinates, and *α* is a set constant.

The repulsion field is the effect of the obstacle on the robot, which prevents the robot from entering the obstacle area. When the distance between the robot and the obstacle is greater than the dangerous distance, the repulsion field is zero, and the robot is moved by the attraction to the target point. When the distance between the robot and the obstacle is less than the safety distance, the repulsion field increases rapidly, and its direction is away from the obstacle. The traditional repulsion field function is defined as

(3) $$\begin{array}{c} U_{\text{rep}}\left(X_{\text{r}}\right)=\left\{\begin{array}{cc} L\times \left(\frac{D_{\text{d}}}{\left|X_{\text{o}}-X_{\text{r}}\right|}\right){}^{\beta }, & \left|X_{\text{o}}-X_{\text{r}}\right|\leq D_{\text{d}},\\ 0, & \left|X_{\text{o}}-X_{\text{r}}\right|>D_{\text{d}}, \end{array}\right. \end{array}$$

where *L* is a constant, **X**_o_ represents the obstacle coordinates, *D*_d_ represents dangerous distance, and *β* is a set constant.

*D*_d_ is usually double of the robot's diameter *D*_r_. In the grid map, the value of *D*_d_ is set by the number of grids. For example, if the robot diameter *D*_r_ is 250 mm and the grid value *D*_g_ is 375 mm, then *D*_d_ is taken to two grids. Suppose the coordinates of the target grid **X**_g_ is (*a*, *b*), the danger zone *R*_d_ can be calculated as

(4) $$\begin{array}{c} R_{\text{d}}=\left\{\left.\left(i,j\right)\;\right|\;i\geq a-2,i\leq a+2,j\geq b-2,j\leq b+2\right\}, \end{array}$$

where (*i*, *j*) are the coordinates of those grids within the hazard distance.

The total potential field *U*_sum_(**X**_r_) is defined as

(5) $$\begin{array}{c} U_{\text{sum}}\left(X_{\text{r}}\right)=U_{\text{attr}}\left(X_{\text{r}}\right)+U_{\text{rep}}\left(X_{\text{r}}\right). \end{array}$$

According to the distribution of attractive field and repulsive field, the magnitude and direction of the force effecting on the robot in each grid can be calculated. When the robot is located in the grid far away from the obstacle, it is only subjected to the attraction pointing to the target. When the distance is closer to the obstacle, the repulsive field increases rapidly, and the robot is subjected the resultant force of attraction and repulsive force, which changes the robot's movement direction.

### 3.2. Improvement about APF

**Figure 2:**
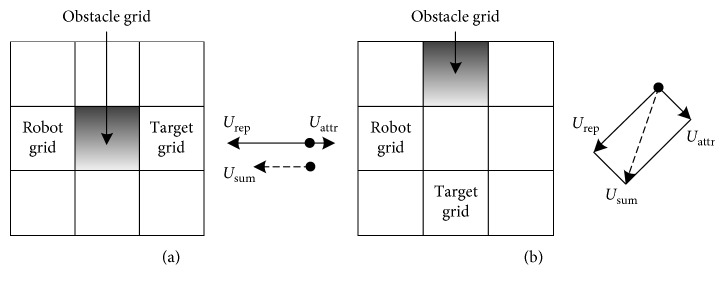
Two kinds of grid traps. (a) The obstacle grid is between the robot grid and target grid. (b) The obstacle grid is not between the robot grid and target grid.

It is easily fallen into a local minimum when using the traditional APF for path planning in the case of the two kinds of gird traps shown in Figure 2. In Figure 2(a), attraction and repulsive force are all along the horizontal direction, and the repulsive force is greater than the gravitational force. So, the resultant force is opposite to the target, and the robot will fall into a dead-end cycle of transverse reciprocating motion. In Figure 2(b), the robot is subjected the attraction force in the upper right of 45° and the repulsive force in the lower right of 45°, and the repulsion is greater than the attraction. Therefore, the robot will move away from the obstacle and the target point.

For these defects, the traditional APF is improved by employing the global environment information of the grid map as follows.

#### 3.2.1. Improving the Attractive Function

For the grid rap shown in Figure 2, the attractive field distribution opposite to that of the conventional potential field is used, which is described as

(6) $$\begin{array}{c} U_{\text{attr}}\left(X_{\text{r}}\right)=\left\{\begin{array}{cc} K\times \left(\frac{S}{\left|X_{\text{r}}-X_{\text{g}}\right|}\right){}^{\alpha }, & \left|X_{\text{r}}-X_{\text{g}}\right|<S,\\ K, & \left|X_{\text{r}}-X_{\text{g}}\right|\geq S, \end{array}\right. \end{array}$$

where *S* is the threshold of the attractive distance.

*S* is generally taken to 1/10 of the grid map length and width. If the value of *S* is 3 and the coordinates of the target grid **X**_g_ is (*c*, *d*), then attractive area *R*_attr_ is

(7) $$\begin{array}{c} R_{\text{attr}}=\left\{\left.\left(i,j\right)\;\right|\;i\geq c-3,i\leq c+3,j\geq d-3,j\leq d+3\right\}, \end{array}$$

where (*i*, *j*) are the coordinates of those grids within the attractive distance.

#### 3.2.2. Improving the Resultant Force of APF

**Figure 3:**
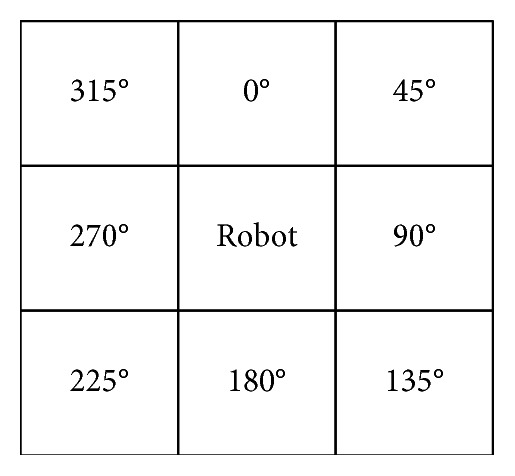
The angle of eight grid neighbors of robot.

There are only eight cases of the robot's transfer directions in the grid map; that is, the resultant force has eight standard directions. The direction of vertically upward is set to 0° and clockwise is the positive direction. The angular distribution of eight standard directions in the grid map is shown in Figure 3.

The angle difference between the resultant force direction and the eight standard directions is calculated respectively. The standard direction with the smallest absolute value of the angle difference is the direction of the resultant force. If the minimum absolute value of the angle difference has two or more standard directions, then the smaller standard direction is adopted as the resultant force direction.

#### 3.2.3. Additional Force

**Figure 4:**
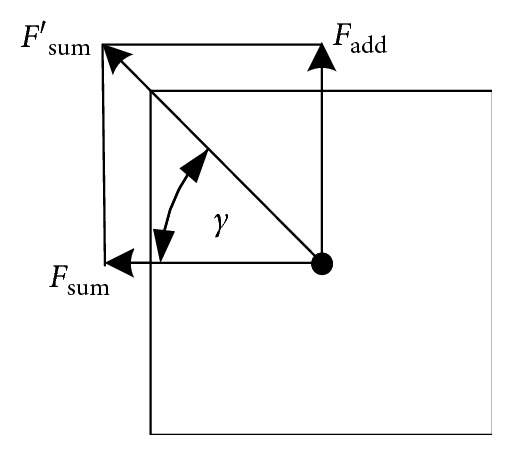
Force analysis of adding additional force.

When the direction of the robot's resultant force is opposite to the direction of the robot's attraction, the robot will fall into an endless loop. At this moment, the robot will be forced out of the endless loop by adding an additional force perpendicular to the resultant force. The force analysis of adding an additional force is shown as Figure 4. In Figure 4, *F*_sum_ is the original resultant force, *F*_add_ is the added additional force, *F*_sum_′ is the new resultant force, and *γ* is the angle between the new resultant force and the original resultant force. To ensure *F*_sum_′ forces the robot to move out of the loop, there must be *γ* > 22.5°. So it should be *F*_add_ > tan  22.5° × *F*_sum_.

## 4. Transition Algorithm of Ant Colony

The improved ACO optimization is divided into two phases. In the prophase, the direction of the resultant force calculated by the improved APF is used to be as inspired factor of ACO. In the anaphase, inspired factor is canceled, and ant colony transition is completely based on pheromone updating.

The paper uses the cellular ant algorithm to plan robot's path, which imitates ants' movement through the moving and iterating of cellular. The transition algorithm of ant colony is designed by pseudorandom proportion rule. The rule uses prior knowledge about the problem and carries out a search with tendentiousness, which forces some ants to choose these grids of nonmaximally probability for searching the other path except the optimal path.

### 4.1. Transition Probability Function

The transition probability function of ant colony cellular automata is given as

(8) $$\begin{array}{c} P_{ij}=\frac{{\left[\tau_{ij}\right]}^{p_{\alpha }}\cdot {\left[\eta_{ij}\right]}^{p_{\beta }}}{\sum_{j\in C_{i}}{\left[\tau_{ij}\right]}^{p_{\alpha }}\cdot {\left[\eta_{ij}\right]}^{p_{\beta }}}, \end{array}$$

where *P*_*ij*_ is comprehensive probability, *η*_*ij*_ is the visibility of arc (*i*, *j*) decided by inspiring factor, *τ*_*ij*_ is the track strength of edge arc (*i*, *j*) which means the number of pheromone on the trajectory, *p*_*α*_ is the relative importance of the trajectory, *p*_*α*_ is the relative importance of the visibility, **C**_*i*_{*c*_0_, *c*_1_,…, *c*_7_} is the neighbor cells set of the cellular automata *c*_*i*_, and *j*=0,1,…, 7.

*η*_*ij*_ represents the visibility between the target location of next step and the current cellular automata. The higher the visibility is, the greater the preference probability is, and the greater *η*_*ij*_ is. In the initial of operation, the value of visibility can be formulized as

(9) $$\begin{array}{c} \eta_{ij}=\left\{\begin{array}{cc} \frac{1}{\left|\langle M_{j},F_{i}\rangle \right|}, & \left|\langle M_{j},F_{i}\rangle \right|>\frac{1}{D},\\ D, & \left|\langle M_{j},F_{i}\rangle \right|\leq \frac{1}{D}, \end{array}\right. \end{array}$$

where the vector **M**_*j*_(*j*=0,1,…, 7) is determined by the cellular automata *c*_*i*_ and its neighbor cells. **F**_*i*_ is the resultant force of the artificial potential field acting upon *c*_*i*_. *D* is the maximum visibility defined in advance. The visibility of the neighbor cells to *c*_*i*_ is determined by the angle between **M**_*j*_ and **F**_*i*_. The smaller the angle is, the higher the visibility is.

To reflect the visibility of neighbor cells in different directions and simplify the algorithm calculation, the visibility is discretized to. According to equation (9), eight-neighbor cells are sorted according to the size of their visibilities. The vector of visibility is defined as **V**{*v*_0_, *v*_2_,…, *v*_7_} and meets ∑_*l*=0_^7^*v*_*l*_=1, and 1 ≥ *v*_0_ ≥ *v*_1_ ≥ *v*_2_ ≥ *v*_3_ ≥ *v*_4_ ≥ *v*_5_ ≥ *v*_6_ ≥ *v*_7_ ≥ 0.

### 4.2. Updating Mechanism of Pheromone

The hybrid strategy of global updating and local updating is presented. The global updating is used to update the number of pheromone for grids in the map when the ant colony finishes one movement cycle. The local updating is used to update the number of pheromone for grids corresponding cellular automata when the ant colony implements the move rules of cellular automata.

The global updating rule of pheromone is defined as

(10) $$\begin{array}{c} \tau_{ij}^{\text{n}}=\rho \cdot \tau_{ij}^{\text{o}}+\underset{k}{\sum }\Delta \tau_{ij}^{k}, \end{array}$$

where *ρ*(0 ≤ *ρ* < 1) is the durability of the trajectory, *τ*_*ij*_^n^ is the new *τ*_*ij*_, *τ*_*ij*_^o^ is the original *τ*_*ij*_, and Δ*τ*_*ij*_^*k*^ is the number of pheromone per unit length on the edge arc (*i*, *j*) of ant *k*.

According to different types of ant colony, Δ*τ*_*ij*_^*k*^ can be set different values. It can be described by threshold mechanism as

(11) $$\begin{array}{c} \Delta \tau_{ij}^{k}=Q=\left\{\begin{array}{cc} Q_{\max}, & N_{n}\leq M_{n},\\ Q_{\min}, & M_{n}<N_{n}\leq S_{n},\\ 0, & N_{n}>S_{n}, \end{array}\right. \end{array}$$

where *Q*_max_ and *Q*_min_ are constants to reflect the track number kept by ants, respectively; *M*_*n*_ is the threshold of node determined by the map information, the starting point, and the end point; *N*_*n*_ is the node number of planned path; and *S*_*n*_ is the maximum number of skipping steps.

The principle of equation (11) is that when *N*_*n*_ < *M*_*n*_, the updating of nodes pheromone will be an optimum global updating and when *M*_*n*_ < *N*_*n*_ ≤ *S*_*n*_, the updating of nodes pheromone will be an suboptimum global updating.

When ants move from the former cellular automata to the next, they will record the coordinate of the cellular automata and update the amount of current cellular automata pheromone. The locally updating mechanism can prevent ants to be lazy, which avoids the algorithm converging to a known current path and giving up finding new paths. The locally updating mechanism can be represented as

(12) $$\begin{array}{c} \tau_{ij}^{\text{n}}=\tau_{ij}^{\text{o}}+\Delta \tau_{ij}^{k}. \end{array}$$

### 4.3. Ant Colony Punishment Mechanism

The comprehensive probability *P*_*ij*_ can be calculated by equation (8). The moving rules of cellular automata can be represented as

(13) $$\begin{array}{c} p\left(i_{t+1}\right)=\left\{\begin{array}{cc} c_{0,t}, & 0\leq R<P_{i0},\\ c_{1,t}, & P_{i1}\leq R<P_{i2},\\ \vdots & \\ c_{7,t}, & P_{i6}\leq R\leq P_{i7}, \end{array}\right. \end{array}$$

where **p**(*i*_*t*+1_) is the position of cellular automata *c*_*i*_ at time *t*+1, *R* is a random integer from 0 to 100, *c*_*n*,*t*_ ∈ **C**_*i*_(*n*=0,1,…, 7), and *P*_*im*_=100 × ∑_*l*=0_^*m*^*P*_*il*_(*m*=0,1,…, 7).

### 4.4. Moving Rules of Cellular Automata

**Figure 5:**
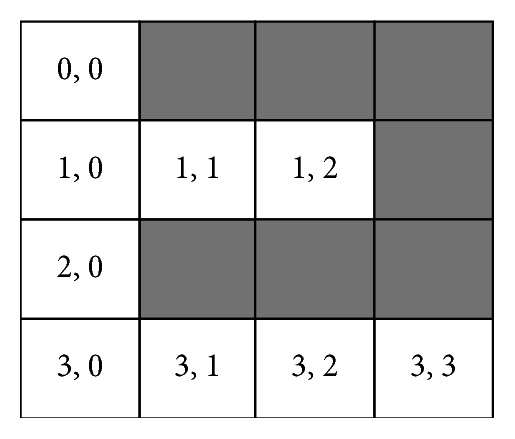
The typical form of a trap grid.

It is possible that there are some trap grids in the grid map. Ants will lose their way when they move into these traps. As shown in Figure 5, it is assumed that the robot starting point is (0, 0) and the target point is (3, 3). The probabilities of ants moving from (0, 0) to (1, 1) and from (1, 1) to (1, 2) are 60% and 100%, respectively. Therefore, a lot of ants will fall into a trap grid.

A punishment mechanism is developed to avoid this trap phenomenon. Firstly, the last five nodes of the current ant are traversed to judge whether these five nodes are the same. Secondly, if these five nodes are the same node, the ant queue is deleted from the active queues and its header is set to 2, and the grid corresponding to the node is treated as an obstacle grid and to be marked to 0. Lastly, the jumping probability table of ants is updated.

## 5. Experiment and Discussion

### 5.1. Simulation and Contrast Experiments

In this paper, experiment platform is VC++ 6.0, the diameter of robot is 250 mm, the size of environment is 12 m × 9 m, and the map's pixel is 640 × 480. The robot's path is planned by the improved APF, the APF-ACO mixed method, the traditional ACO, and the traditional APF, respectively. The feasibility, advantage, and disadvantage of our algorithm are verified by comparing with other three algorithms from the planning path's time-consuming, number of node, turning angle, etc.

1. Set up the map lattice model. We set the safety distance is 125 mm, *ϕ*=*λ*=1, and *φ*=0. The grid size is 375 mm × 375 mm calculated by equation (1), and the size of the grid is 32 × 24.
2. Set up the potential field in the grid map according to the improved APF. According to the target and obstacle information in the grid map. First, calculate the force of each grid and store them in the corresponding memories. Then, traverse all grids and compare the direction of the resultant force with the direction of attraction. Finally, if the direction of resultant force is opposite to the direction of attraction, the additional force is added to change the direction of the resultant force. We set *α*=*β*=1, *K*=*L*=1, *S*=2, *D*_d_=1, and *F*_add_=0.5 × *F*_sum_.
3. Initialize the pheromone table and calculate the visibility of eight-neighbor cell according to the orientations of grid determined by step 2. We set the initial values of the pheromone of all nonobstacle grids and all obstacle grids to 12.5 and 0, respectively. At the same time, we set **V**={0.4, 0.2, 0.2, 0.08, 0.08, 0.02, 0.02, 0}, *p*_*α*_=2, *p*_*β*_=1, *ρ*=0.8, *Q*_max_=160, *Q*_min_=25, *M*_*n*_=50, and *S*_*n*_=100. So, the pheromone table of jump probability of ants can be got.
4. Place 1000 ants to plan the path by the improved ACO. Cellular automatas move according to the table of jump probability. The pheromone table and the path nodes of ants can be updated or recorded synchronously. The process is repeated until the shortest path is outputted. We get the ants nodes the shortest planned path and computational time.
5. Run the improved APF to plan the path based on the direction of resultant force by step 2. The process is repeated 100 times, and then output the nodes of the planned path and computational time.
6. Run the traditional ACO to plan the path. We set the visibilities *v*_*i*_=0.125(*i*=0,1,…, 7), the amount of ants to 1000, and other parameters same as step 3. Finally, we get the planning results, including the ant nodes of the shortest path and computational time.

**Figure 6:**
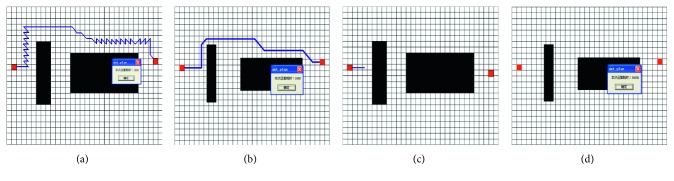
The experimental results. (a) The path planned by improved APF; (b) APF-ACO mixed method; (c) the traditional APF; (d) the traditional ACO.

The paths planned by four algorithms are shown in Figure 6.

The parameters of calculating and path are given in Table 1.

The traditional APF falls into an endless loop at the beginning of the path and fails to reach the target point after 100 repeated experiments. The improved APF can avoid obstacles and takes 1500 ms to plan the path from the starting point to the target point. This shows that the improved APF can effectively solve the problem of local minimum. But, the planned path is not satisfactory because it has the path length error of 96.9% and the turning angle error of 1600%.

Using the traditional ACO, only a few ants reach the desired position within the set 100 steps. So, the traditional ACO cannot get a convergence result to form a path within the given time of 30000 ms. There are various reasons for the failure, among which the main reason is that the initial values of the visibilities *v*_*i*_(*i*=0,1,…, 7) are no heuristic for ant colony searching. In fact, we can get satisfactory results in some experiments based on a suitable set of **V**. But, the problem is that we do not know how to set these parameters properly.

Our mixed algorithm can obtain a more satisfactory result. Compared to the improved APF, although the mixed algorithm takes more than 390 ms, it plans out a better path which reduces unnecessary turning and redundant paths and has the path length error of 10.3% and the angle error of 100%. Compared to the traditional ACO, the mixed algorithm improves the convergence speed and gets a convergence path with the same *p*_*α*_, *p*_*β*_, and initial pheromone. As a result, this mixed algorithm combines the advantages of APF and ACO and has better performance of path planning.

### 5.2. Mobile Robot Motion Experiment

The purpose of mobile robot experiments is to verify the APF-ACO mixed algorithm adaptability to different tasks. In this paper, experiments are designed to mobile robot's path planning and path tracking from different start points to different end points in a same obstacle environment.

**Figure 7:**
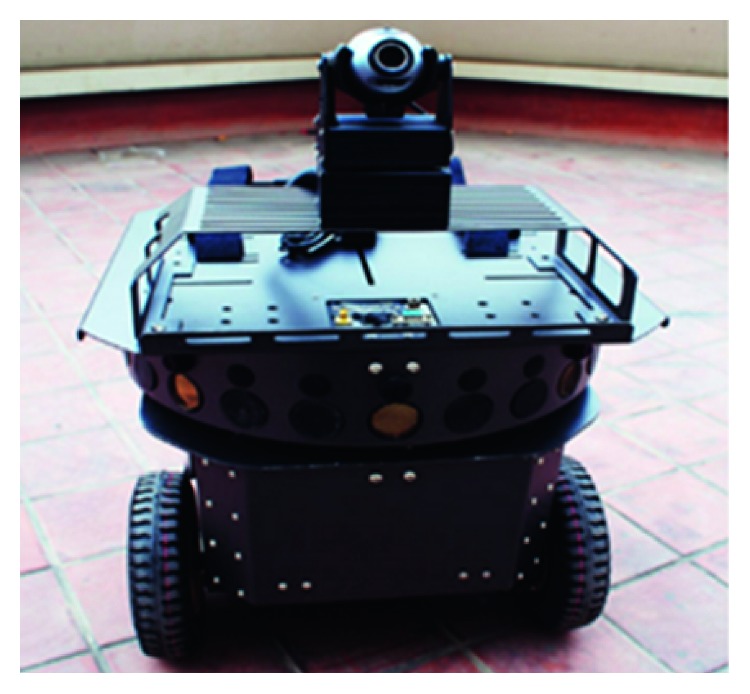
The image of Traveler II robot.

The mobile robot used in experiments is Traveler II as shown in Figure 7, which can be considered as a cylinder with a diameter of 250 mm.

**Figure 8:**
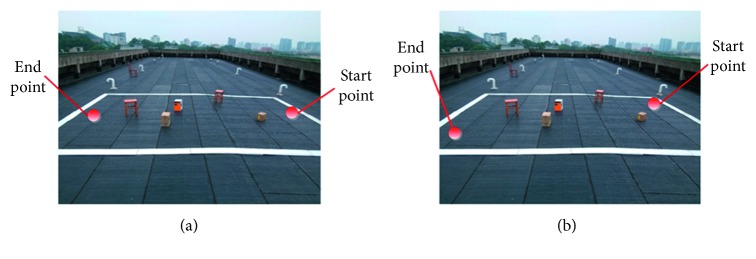
The experimental environment and two kinds of start point and end point. (a) The setting points (I), its start point coordinates and end point coordinates are (187.5, 4125) and (187.5, 8625), respectively; (b) the setting points II, its start point coordinates and end point coordinates are (11625, 4125) and (11625, 187.5), respectively.

The mobile robot's experimental environment with the size of 12 m × 9 m and two sets mobile robot's start and end points are designed as shown in Figure 8.

A comprehensive index for the planned path is presented to value the merits and effectiveness of the proposed APF-ACO mixed algorithm. The index is defined as

(14) $$\begin{array}{c} \text{ST}=\sigma \times \text{Len}+\varsigma \times \text{Tangle}, \end{array}$$

where Len is the length of planned path, Tangle is the turning angle of planned path, and *σ* and *ς* are weighted coefficients, and *σ*+*ς*=1. *σ* is taken a larger value than *ς* if the path length is paid more attention is concerned. Otherwise, *ς* is taken a larger value than *σ*.

**Figure 9:**
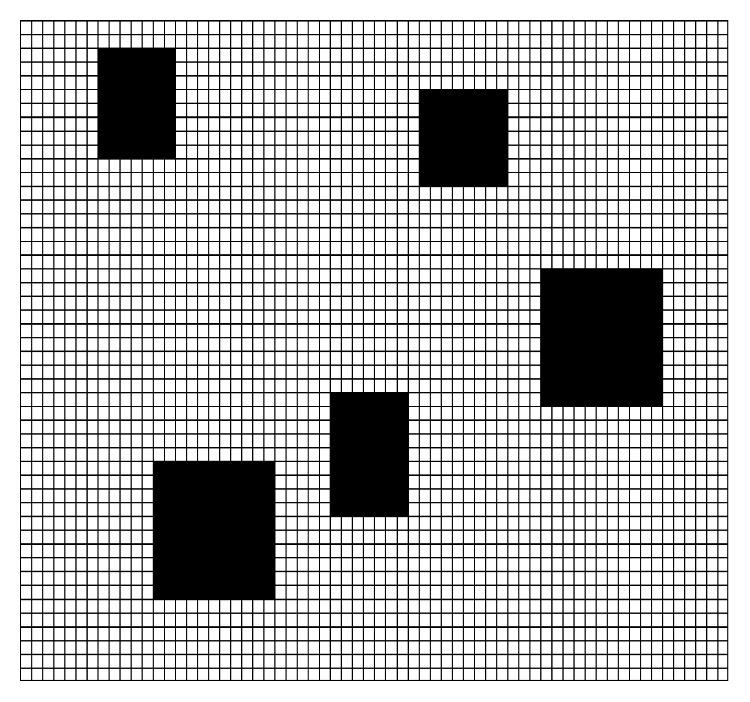
Raster map for barrier environment.

**Figure 10:**
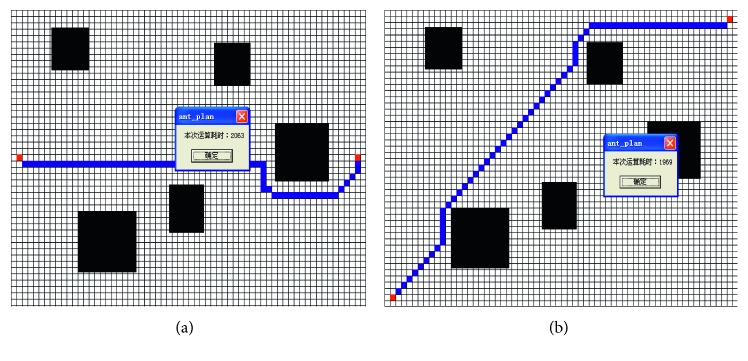
Path planning results. (a) The path planned by APF-ACO algorithm for the setting points I and (b) APF-ACO algorithm for the setting points II.

The experimental steps are as follows:

1. *Grid modeling*. This paper sets the size of grid map to 640 × 480 (pixels), *ϕ*=*λ*=0.5, and *φ*=0. Then the size of a single grid is 187.5 mm × 187.5 mm, and the ratio of the grid size and real environment size is 4 : 75. So, the mobile robot's environment shown by Figure 8 can be rasterized to grid map as shown in Figure 9. According the grip size of 187.5 mm, the coordinates of two sets of start and end points in Figure 9 are transferred to (1, 22), (62, 22) and (1, 46), (62, 1), respectively.
2. *Establishing the potential field*. This paper sets *α*=*β*=2*K*=*L*=1*S*=3*D*_d_=2, and *F*_add_=0.5 × *F*_sum_. According to the potential field and the information of grid map, we can calculate the resultant force of each grid and its direction, and the information is stored.
3. *Path planning*. We set **V**={0.5, 0.18, 0.18, 0.05, 0.05, 0.02, 0.02, 0,0}, *p*_*α*_=2, *p*_*β*_=1*ρ*=0.8, *Q*_max_=160, and *Q*_min_=25 and placed 1000 ants in the grid map. Lastly, the optimum path obtained by APF-ACO algorithm is shown in Figure 10.
4. *Mobile robot motion control*. Firstly, we obtain the sequences of nodes in grid map of the path planned by the APF-ACO algorithm. Secondly, the coordinate transformation of the next node is processed from the grid map to the environment map. Then, the mobile robot's displacement length and the turning angle between the current node and the next node can be calculated. So, according to these motion parameters and control laws, the function of the left and right wheels' velocity and time can be determined. Finally, the mobile robot is controlled to move until it reaches the last node by the function.

**Figure 11:**
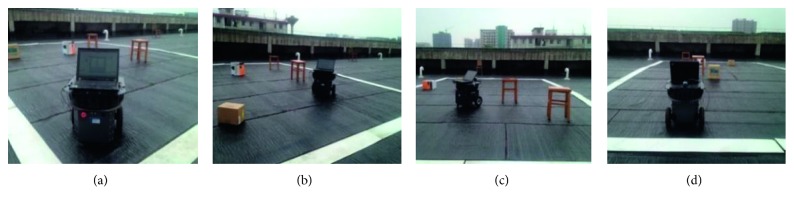
The images of moving process of mobile robot.

Figure 11 shows the moving process of the mobile robot. The motion results show the mobile can move from the start point to the end point along a smooth and nonobstacles path.

Table 2 gives some results of the algorithm execution process and planning path. The data show the APF-ACO algorithm can plan a higher quality path.

Further, the error rate of the path length *e*_*l*_ is defined as

(15) $$\begin{array}{c} e_{l}=\frac{\left|l_{\text{p}}-l_{\text{a}}\right|}{l_{\text{p}}}, \end{array}$$

where *l*_a_ is the mobile robot's actual path length and *l*_p_ is theoretical movement distance.

The error rate of consumption time *e*_*t*_ is defined as

(16) $$\begin{array}{c} e_{t}=\frac{\left|t_{\text{p}}-t_{\text{a}}\right|}{t_{\text{p}}}, \end{array}$$

where *t*_a_ is the actual consumption time and *t*_p_ is the theoretical consumption time.

The error rate of path length and the error rate of consumption time are shown in Table 3, which shows that the error rate of path length and the error rate of consumption time will increase correspondingly with the increase of the path nodes. The maximum error rate of path length is 6.58%, and the maximum value of the error rate of time is 13.97%. Considering the accumulated error of the inertial sensor of the mobile robot, the error is within the expected range.

## 6. Conclusion

This paper presents a hybrid path planning mixed of an improved APF and an improved ACO for mobile robot based on grid map. The main improvements are as follows:

1. The improvements about APF: Firstly, to avoid grid traps, the attraction is calculated by the reciprocal of the distance between the current position and the target position. Secondly, to simplify the calculation, the direction of resultant force is separated into 8 directions based on the current grid. Thirdly, an additional force perpendicular to the direction of the resultant force is added to form a new resultant force when the robot falls into an endless loop.
2. The improvements about ACO: The process of optimization is divided into two phases. In the prophase, the direction of the resultant force obtained by the improved APF is used as the inspired factors, which leads ant colony to move in a directional manner. In the anaphase, the inspired factors are canceled, and ant colony transition is completely based on pheromone updating, which can overcome the inertia of the ant colony and forces them to explore a new and better path.

In the grid environment, the planning result by the improved APF is employed as the optimizing foundation of ACO. The method overcomes some drawbacks of APF such as goal being nonreachable and easily falling into the extreme point, etc., and improves the optimization effect and the rate of convergence of ACO. The simulation experiments and mobile robot environment experiments verify that the method has stronger stability and environmental adaptability.

## Figures and Tables

**Table 1:** Test results of robot path planning by three algorithms.

Algorithm	Time-consuming (ms)	Grid number of path	Path length (the number of grids)	Turning angle (rad)
APF-ACO	1890	32	34	2*π*
Improved APF	1500	57	65	27*π*
Traditional ACO	30000	0	0	0
Traditional APF	∞	3	3	0

**Table 2:** Process and result data.

Items	Setting points I	Setting points II
Time-consuming (ms)	2063	1969
Numbers of path nodes	66	68
Path length (unit grid)	69	84
Turning angle (radian)	2*π*	1.5*π*
Comprehensive indicator (ST)	9.61	10.2
Robotic exercise time (s)	147	155
Robot moving distance (mm)	13788	16661

**Table 3:** The results of path tracking.

Items	Experiment of setting points I	Experiment of setting points II
*e*_*l*_ (%)	6.58	5.78
*e*_*t*_ (%)	11.36	13.97

## Data Availability

The research library related to the dissertation in this study is established in https://github.com/glchenwhut/research.git, where you can access the folders and find experimental data and lists.
